# A Bibliometric Analysis of Publication Patterns in Pediatric Neurology

**DOI:** 10.3389/fped.2022.753554

**Published:** 2022-06-16

**Authors:** Mitch Wilson, Margaret Sampson, Nick Barrowman, Ewa Sucha, Asif Doja

**Affiliations:** ^1^Department of Neurology, Beth Israel Deaconess Medical Center, Boston, MA, United States; ^2^Children’s Hospital of Eastern Ontario, Ottawa, ON, Canada; ^3^Children’s Hospital of Eastern Ontario Research Institute, Ottawa, ON, Canada

**Keywords:** bibliometrics, publication patterns, subspecialties, journals, pediatric neurology, neurology

## Abstract

**Purpose:**

To examine the publication patterns of pediatric neurology articles in general pediatric, general neurology, and neurology subspecialty journals using a bibliometric approach.

**Methods:**

The top 5 journals in general pediatrics, general neurology and neurology subspecialties were identified using the 2017 Journal Citations Report (JCR). For general pediatric journals, we selected 4 pediatric subspecialties for comparison of publication patterns with neurology: immunology, endocrinology, gastroenterology, and respirology. For general neurology and neurology subspecialty journals, we searched both the top 5 neurology and neurology subspecialty journals for pediatric articles. Using Ovid Medline, we identified articles published between 2009–2017.

**Results:**

With regards to child neurology-based articles, 1501 were published in general pediatrics journals, 643 in general neurology journals and 685 in neurology subspecialty journals. Examination of the top pediatric journals revealed that *Pediatrics* published the most neurology-based articles. Neurology-based studies were published more frequently than other pediatric subspecialty studies. Of the top general neurology *Neurology* published the most child neurology-based articles, while *Epilepsia* published the most child neurology-based articles out of neurology subspecialty journals. Cohort studies were the most frequent study type across all journals.

**Conclusion:**

Our study revealed that child neurology articles are published more often in pediatric journals as opposed to general neurology and neurology subspecialty journals. We also found that in general pediatric journals, neurology-based articles are published more frequently compared to other specialties. Our results provide guidance to authors when considering submission of their pediatric neurology research.

## Introduction

Research in the field of pediatric neurology continues to grow over time with an increasing number of journals in which researchers can publish their work. Peer reviewed pediatric neurology focused research articles are published in a variety of journals, including general pediatric journals, neurology journals, and neurology subspecialty journals. At present, our knowledge regarding the precise publication patterns of pediatric neurology articles in such journals is limited.

One way to explore this is through bibliometric analysis, which can provide data concerning productivity rates, publication patterns, and publication characteristics. Bibliometric research provides statistical descriptions of publications and is based, in part, on the premise that the published literature of a field embodies the field’s knowledge (1, 2). It employs computerized analytic techniques and uses the individual publication as the unit of analysis, drawing data from sources such as MEDLINE, Google Scholar, and Journal Citation Reports (JCR) (3). Articles’ metadata—including the indexing terms applied by the National Library of Medicine (NLM) and provided in MEDLINE—are retrieved and analyzed rather than articles’ full text, which does not need to be obtained or examined. Bibliometric methods have been used to explore the productivity of researchers, institutions, and countries within given subject areas; to examine research trends and emphases in various disciplines; and to guide policy decision making (4–7).

The purpose of our study was to examine the publication patterns of pediatric neurology-based research, utilizing a bibliometric approach. Specifically, we sought to examine the bibliometric patterns of the following: (1) pediatric neurology-based articles published in general pediatric journals; (2) pediatric neurology-based articles published in general neurology journals; and (3) pediatric neurology -based articles published in neurology subspecialty journals.

## Materials and Methods

### Journal Selection

#### Pediatric Journals

The top 5 general pediatric journals were identified using the Journal Citations Report (JCR) by Clarivate Analytics. The JCR generates a list of journals ranked by impact factor which is a measure reflecting the average number of citations to articles published in that journal. The Journals data were filtered by (1) Selected Categories: “PEDIATRIC,” (2) Selected JCR Year: 2017, (3) Selected Editions: Science Citation Index Expanded (SCIE), and (4) Selected Category Scheme: Web of Science (WoS). After generating our selected list of journals from the JCR we then identified the 5 journals with the highest impact factors meeting the following criteria: (1) the journal must be a general pediatric journal (i.e., not a pediatric subspecialty journal), and (2) the journal must publish primary research reports (i.e., not exclusively review articles). We then obtained each journal’s 2017 impact factor (8) which is a measure reflecting the average number of citations to articles published in that journal.

We sought to compare the publication patterns seen in pediatric neurology against 4 other pediatric subspecialties. To establish these 4 additional disciplines we examined the JCR categories and selected the 4 general medicine specialties with the highest number of total citations in the JCR 2017 report that also met the following criteria: (1) the specialty must not focus exclusively on the adult age group (i.e., geriatrics would be excluded), (2) articles in the specialty must not consist predominantly of pathology or basic science research.

Some articles could be indexed in our search by more than one specialty. We acknowledged that this would result in duplicate publications being included in our study, however since there is no fair and objective way to choose one specialty over another for a publication, we elected to include all duplicate versions of an article that were indexed for different specialties.

#### Neurology Journals

The top 5 general neurology journals were identified using the JCR. The Journals data was filtered by: (1) Selected Categories: “CLINICAL NEUROLOGY,” (2) Selected JCR Year: 2017, (3) Selected Editions: SCIE, and (4) Selected Category Scheme: WoS. After generating our selected list of journals from the JCR we then identified the 5 journals with the highest impact factors meeting the following criteria: (1) the journal must be a general neurology journal (i.e., not a neurology subspecialty journal), (2) the journal must publish primary research (not exclusively review articles), (3) the journal must not publish exclusively adult neurology articles, and (4) the journal must not publish exclusively pathology, neuropathology or basic science research.

#### Neurology Subspecialty Journals

The top 5 neurology subspecialty journals were identified using the JCR. The Journals data was filtered by (1) Selected Categories: “CLINICAL NEUROLOGY,” (2) Selected JCR Year: 2017, (3) Selected Editions: SCIE, and (4) Selected Category Scheme: WoS. After generating our selected list of journals from the JCR we then identified the 5 journals with the highest impact factors meeting the following criteria: (1) the journal must be a subspecialty neurology journal (i.e., not a general neurology journal), (2) the journal must publish primary research (not exclusively review articles), (3) the journal must not publish exclusively adult neurology articles, (4) the journal must not publish exclusively pathology, neuropathology or basic science research, and (5) the focus of the journal must not span multiple disciplines (e.g., Pain, Sleep, Neuro-oncology).

For all journals, where not evident from the journal title, eligibility was determined by examining the journal instructions to authors or statement of the journal scope. If eligibility was still unclear, it was resolved by exploratory searching followed by a consensus decision of the 3 investigators.

### Bibliometric Search Strategy

#### Part 1: Pediatric Journals

On April 25, 2019, Using Ovid MEDLINE, we searched the top five general pediatric journals for articles published between 2009 and 2017 that were indexed with the following MeSH (Medical subject headings) terms: *nervous system diseases, immune system diseases, endocrine system diseases, gastrointestinal diseases*, and *respiratory tract diseases.*

#### Part 2 and 3: Neurology and Neurology Subspecialty Journals

On May 8, 2019, Using Ovid MEDLINE, we searched the 5 general neurology journals and 5 neurology subspecialty journals for articles published between 2009 and 2017. We limited our search to pediatric articles. We also conducted a search in which we did not limit the search to pediatric articles to measure the proportion of total articles that were indexed as pediatric.

For all 3 searches (pediatric, neurology and neurology subspecialty), we further restricted the search to records with published abstracts. We also excluded commentaries, editorials, historical articles, and letters. Records were downloaded into Reference Manager version 11 (Thomson Reuters, New York, NY, United States). Please see Supplementary Appendix 1 for full details of the search strategy.

### Study Designs

We included in our search the MEDLINE classification for the following 8 study designs: randomized controlled trials (RCT), clinical trials, systematic reviews, case reports, cohort, cross-sectional, case-control, and comparative. A hierarchical filtering process was used in Ovid to ensure all study types were mutually exclusive and that each article was only indexed for one study type.

### Search Strategy Development

The search strategies were developed by the second author (MS).

### Review of Classification Accuracy

We reviewed a random 5% sample of the records to assess classification accuracy for specialty and study type for all 3 bibliometric searches. In the neurology and neurology subspecialty searches, we also assessed the accuracy of articles being correctly classified as pediatric.

### Statistical Analysis

For comparison of neurology and other specialties as well as for comparison of study type in pediatric journals, we constructed models for the number of publications by year, journal, and subspecialty and for the number of publications by year, journal, and study type, respectively. In both cases, Poisson models were initially considered; however, due to the presence of overdispersion these models were replaced by quasi-Poisson models. The starting point were models including main effects (for year, journal, and subspecialty in the first comparison and for year, journal, and study type in the second comparison) as well as all two-way interactions. When the evidence for an interaction was weak (*p* > 0.10), that interaction was removed, and the reduced model was fitted. Likelihood ratio test was used to compare full and reduced models. Based on the final models, estimated marginal means by year, journal, and subspecialty and by year, journal, and study type, respectively were computed.

Next, contrasts between each subspecialty and neurology within journals (comparison of neurology and other specialties) were estimated. The contrasts were then averaged over years, and displayed as ratios, together with 95% confidence intervals adjusted for multiple testing using the multivariate *t*-distribution method (9).

### Ethical Approval

The need for ethical approval was waived by the Children’s Hospital of Eastern Ontario Research Institute Ethics Board, as this was a purely bibliometric study.

## Results

### Pediatric Journals

The JCR 2017 report revealed that the five general pediatric journals with the 5 highest impact factors were: *The Journal of the American Medical Association (JAMA) Pediatrics* (10.8), *Pediatrics* (5.5), *Archives of Disease in Childhood Fetal and Neonatal Edition* (4.0), *Journal of Pediatrics* (3.7), and *Pediatric Research* (3.1) (Table 1). Note that *JAMA Pediatrics* continued *Archives of Pediatrics and Adolescent Medicine*, the name change occurred during our observation period. We searched both, combining results and will refer to this set as *JAMA Pediatrics*. The 4 selected specialties used for comparison with neurology were immunology, endocrinology, gastroenterology, and respirology.

**TABLE 1:** Productivity of the top 5 pediatrics journals in the specialty of neurology, including distribution by journal and by study design.

	JAMA pediatrics	Pediatrics	Archives of disease in childhood fetal and neonatal edition	Journal of pediatrics	Pediatric research	Total
JCR Rank	1	3	7	8	16	
Journal impact factor	10.8	5.5	4.0	3.7	3.1	
Study design						
RCT	11	76	14	34	8	**143**
Clinical trials	0	5	0	14	0	**19**
Systematic review	11	39	2	0	0	**52**
Case reports	0	184	4	47	8	**243**
Cohort	60	343	50	334	46	**833**
Cross-sectional	5	50	0	32	1	**88**
Case-control	5	12	2	25	22	**66**
Comparative	3	21	4	5	24	**57**
Total	**95**	**730**	**76**	**491**	**109**	

*The bold values are simply the totals of the rows/columns.*

Our bibliometric search yielded 4,239 publications, of which 1,501 were in neurology; see Table 1 for a summary of distribution of these 1,501 neurology publications in the top 5 general pediatric journals. Table 1 also summarizes the type of study design of neurology articles published in the top 5 general pediatric journals. The number of publications in the other 4 specialties was: immunology (715), endocrinology (394), gastroenterology (467), and respirology (1,162) (Table 2). The distribution of publications for each study type by specialty is provided in Table 2.

**TABLE 2:** Distribution of publications in pediatric journals in each of the 5 specialties by journal and by study design.

	Neurology	Immunology	Endocrinology	Gastroenterology	Respirology	Total
Journal						
JAMA pediatrics	95	77	32	28	93	**325**
Pediatrics	730	358	137	190	500	**1915**
Archives of disease in childhood fetal and neonatal edition	76	6	8	17	90	**197**
Journal of pediatrics	491	218	193	200	390	**1492**
Pediatric research	109	56	24	32	89	**310**
Total	**1501**	**715**	**394**	**467**	**1162**	

Study design						
RCT	143	84	26	57	215	**525**
Clinical trials	19	12	9	12	21	**73**
Systematic reviews	52	25	10	19	45	**151**
Case reports	243	153	86	59	125	**666**
Cohort	833	307	194	258	578	**1893**
Cross-sectional	88	66	39	18	62	**273**
Case-control	66	37	17	31	57	**208**
Comparative	57	31	13	13	59	**173**

*The bold values are simply the totals of the rows/columns.*

### Comparison of the Yearly Number of Publications in Neurology and Other Specialties

**FIGURE 1:**
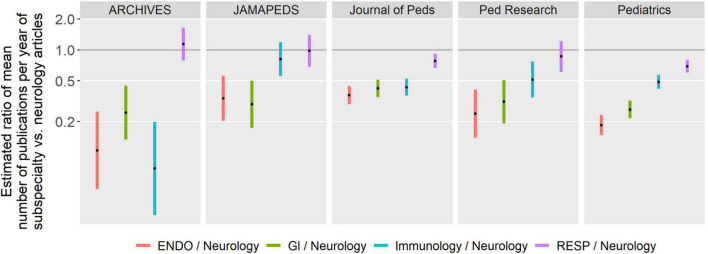
Estimated ratio of number of publications per year for each subspecialty compared to neurology within each journal (*N* = 250 combinations of year, journal, subspecialty). Estimates are shown with 95% confidence intervals adjusted for multiple testing. ENDO, Endocrinology; GI, Gastroenterology; RESP, Respirology.

Figure 1 describes the estimated ratio of mean number of publications per year of subspecialty versus neurology articles. The length of the bars indicate 95% confidence intervals for the estimates. The position of the estimate (the dot in the middle of the bars) should be interpreted in relation to “1.” If the value is below 1, then in a given journal, the number of publications in given a specialty was lower than in the reference specialty (Neurology). If the estimate is above 1, then the number of publications in a given specialty was higher than in Neurology. In all journals considered, there was a greater number of publications in neurology than other specialties with the exception of the number of respiratory-focused publications in *Archives of Disease in Childhood Fetal and Neonatal Edition, JAMA Pediatrics* and *Pediatric Research* and the number of immunology-focused publications in *JAMA Pediatrics* being comparable to neurology.

### Neurology Journals

According to the JCR 2017 report the five general neurology journals with the 5 highest impact factors were: *Lancet Neurology* (27.1), *JAMA Neurology* (11.5), *Brain* (10.8), *Annals of Neurology* (10.3), and *Neurology* (8.1).

When our bibliometric search was performed without a child limit, 5,278 records were identified. After restricting to pediatric articles our search produced 643 records, and thus the proportion of all articles that were indexed as pediatric was 12.2%. Table 3 outlines the distribution of pediatric neurology focused articles in the top five general neurology journals, as well as the study types.

**TABLE 3:** Productivity of the top 5 neurology journals with distribution by journal and by study design.

	Lancet neurology	JAMA neurology	Brain	Annals of neurology	Neurology	Total
JCR rank	1	5	6	8	13	
Journal impact factor	27.1	18.3	13.5	10.42	9.901	
Study design						
RCT	9	1	2	8	25	**45**
Clinical trials	6	0	1	4	6	**17**
Systematic review	1	2	0	0	4	**7**
Case reports	0	9	18	10	45	**82**
Cohort	20	28	55	57	255	**415**
Cross-sectional	2	0	6	0	16	**24**
Case-control	1	2	10	5	12	**30**
Comparative	9	1	2	8	25	**45**
Total	**41**	**42**	**96**	**92**	**372**	

*The bold values are simply the totals of the rows/columns.*

### Neurology Subspecialty Journals

According to the JCR 2017 report the five neurology subspecialty journals with the 5 highest impact factors were: *Movement Disorders* (8.3), *Stroke* (6.2), *Multiple Sclerosis Journal* (5.3), *Epilepsia* (5.1), and *Journal of Stroke* (4.8).

When our bibliometric search was performed without a child limit, 5,513 records were identified. After restricting to pediatric articles our search produced 685 records, and thus the proportion of all articles that were indexed as pediatric was 12.4%. Table 4 outlines the distribution of pediatric neurology focused articles in the top five subspecialty neurology journals, as well as the study types.

**TABLE 4:** Productivity of the top 5 neurology subspecialty journals with distribution by journal and by study design.

	Movement disorders	Stroke	Multiple sclerosis journal	Epilepsia	Journal of stroke	Total
JCR rank	11	16	22	24	28	
Journal impact factor	8.3	6.2	5.3	5.1	4.8	
Study design						
RCT	2	3	0	35	0	**40**
Clinical trials	3	6	0	12	0	**21**
Systematic review	3	0	0	16	0	**19**
Case reports	10	3	0	37	0	**50**
Cohort	28	63	0	355	0	**446**
Cross-sectional	2	3	0	13	0	**18**
Case-control	5	3	0	18	0	**26**
Comparative	1	4	0	60	0	**65**
Total	**54**	**85**	**0**	**546**	**0**	

*The bold values are simply the totals of the rows/columns.*

### Review of Classification Accuracy

Of the 4,239 pediatric journal records, 212 (5%) were reviewed. Accuracy was 94.8% by specialty and 97.6% by study design. Of the 643 neurology journal records, 32 (5%) were reviewed. Accuracy was 100% by specialty and 93.8% by study design, and 93.9% of records were accurately classified as pediatric. Of the 685 neurology subspecialty journal records, 34 (5%) were reviewed. Accuracy was 100% by specialty and 100% by study design, and 100% of records were accurately classified as pediatric.

## Discussion

Our results indicate that *Pediatrics* and *Journal of Pediatrics* publish the majority of neurology publications in pediatric journals. Pediatric journals published more neurology articles than for other specialties. Cohort studies accounted for a significant portion (55%) of all neurology publications, while RCTs accounted for 10%.

Among neurology journals, the majority of pediatric articles were published in the journal *Neurology.* Similar to pediatric journals, cohort studies were the most common study type. RCTs represented only 7% of all studies. This is in contrast to adult neurology publications in general medicine journals (GMJs) where RCTs were the most commonly employed study design (48%) and cohort studies were less common (30%) (10). The paucity of published pediatric RCTs is not a novel finding (11). There are multiple reasons as to why there are so few RCTs in the pediatric literature. For one, diseases that are unique to pediatric populations are relatively rare compared to diseases of adulthood and so the cost of conducting expensive clinical trials can be difficult to justify. There are important ethical and considerations when enrolling children in RCTs such as obtaining proxy consent from caregivers and providing financial incentives to caregivers (12). Concerns regarding acceptability – whereby one group receives treatment and another group does not - are of particular importance in children and likely limit the number of conducted and subsequently published pediatric RCTs.

Among neurology subspecialty journals, the vast majority of pediatric articles were published in *Epilepsia*. This is not surprising given that the other journals are focused on disorders that primarily, if not almost exclusively, affect adults (e.g., stroke, multiple sclerosis, Parkinson’s Disease). Epilepsy, conversely, is more common in childhood with incidence rates ranging from 0.5 to 8 per 1,000 person-years (13, 14). In adults, the incidence rate of epilepsy is highest in older adults, ranging from 1 to 3 per 1000 person-years, and is estimated to be two to six times higher than in younger adults (15).

In the pediatric and neurology journals, there is no clear association between a higher journal impact factor (JIF) and greater number of publications. For instance, the pediatric journals with the 2nd and 5th highest impact factors (*Pediatrics* and *Journal of Pediatrics*) had by far the greatest number of publications, much more than *JAMA Pediatrics* – the pediatric journal with the highest JIF. This trend is in opposition to that seen in neurology articles published in general medical journals whereby *New England Journal of Medicine* – the journal with the highest impact factor - published the most neurology articles (10). Within neurology journals, there is an inverse relationship between number of publications in our sample and impact factor. With decreasing impact factors, authors essentially have more choice. While they could submit to *Journal of Pediatrics*, with an impact factor of 3.7, they could also choose to submit to a neurology specific journal with a similar impact factor.

### Limitations

Inherent to any bibliometric approach, the major limitation of our analysis was that we did not examine individual article records, except those in the random sample we examined to verify the accuracy of indexing. Rather, we relied on indexing in MEDLINE for classifications. Nonetheless, as we previously mentioned, the accuracy of the 5% sample we manually verified was very high and thus we can be confident that the results achieved through bibliometric methods are a true reflection of the specific body of literature.

Our results provide some guidance to authors regarding where they may wish to consider submitting their pediatric neurology research. Prospective authors will be able to incorporate the results into their decision making, based on the intended audience, the perceived impact of the research and the study design utilized.

## Data Availability Statement

The original contributions presented in this study are included in the article/Supplementary Material, further inquiries can be directed to the corresponding author.

## Author Contributions

MW, MS, NB, ES, and AD contributed substantially to the conception or design of the work, the acquisition, analysis, interpretation of data for the work, drafted the work or revised it critically for important intellectual content, gave final approval of the version to be published, and are in agreement to be accountable for all aspects of the work in ensuring that questions related to the accuracy or integrity of any part of the work are appropriately investigated and resolved. All authors contributed to the article and approved the submitted version.

## Conflict of Interest

The authors declare that the research was conducted in the absence of any commercial or financial relationships that could be construed as a potential conflict of interest.

## Publisher’s Note

All claims expressed in this article are solely those of the authors and do not necessarily represent those of their affiliated organizations, or those of the publisher, the editors and the reviewers. Any product that may be evaluated in this article, or claim that may be made by its manufacturer, is not guaranteed or endorsed by the publisher.
