# Supplementary material for: A Bibliometric Analysis of Publication Patterns in Pediatric Neurology
Source: Front Pediatr. 2022 Jun 16;10:753554. doi: 10.3389/fped.2022.753554 (PMC9243535; doi:10.3389/fped.2022.753554)
Supplement: Supplementary file 1 [file Data_Sheet_1.PDF]

Ovid®

Wolters Kluwer

My Account Support & Training Help Feedback Logged in as M Sampson at CHEO Library Logoff
Ask a Question

Search Journals Books My Workspace Visible Body EBP Tools Amirsys Multimedia

▼ **Search History** (110) View Saved

# ▲	Searches	Results	Type	Actions	Annotations
1	exp Nervous System Diseases/	2372996	Advanced	Display Results | More	
2	limit 1 to (abstracts and last 10 years)	663764	Advanced	Display Results | More	
3	(jama pediatrics or "archives of pediatrics & adolescent medicine").jn.	6634	Advanced	Display Results | More	
4	pediatrics.jn.	35256	Advanced	Display Results | More	
5	archives of disease in childhood fetal & neonatal edition.jn.	3545	Advanced	Display Results | More	
6	journal of pediatrics.jn.	31758	Advanced	Display Results | More	
7	"pediatric research".jn.	11732	Advanced	Display Results | More	
8	2 and (or/3-7)	2269	Advanced	Display Results | More	
9	remove duplicates from 8	2268	Advanced	Display Results | More	
10	limit 9 to (comment or editorial or festschrift or historical article or letter)	10	Advanced	Display Results | More	
11	9 not 10	2258	Advanced	Display Results | More	
12	limit 11 to child	1023	Advanced	Display Results | More	
13	limit 11 to adult	194	Advanced	Display Results | More	
14	limit 11 to "qualitative studies (optimized)"	220	Advanced	Display Results | More	
15	limit 11 to clinical trial, all	181	Advanced	Display Results | More	
16	limit 11 to randomized controlled trial	149	Advanced	Display Results | More	
17	limit 11 to systematic reviews	115	Advanced	Display Results | More	
18	limit 11 to comparative study	263	Advanced	Display Results | More	
19	limit 11 to evaluation studies	44	Advanced	Display Results | More	
20	11 and Program Evaluation/	11	Advanced	Display Results | More	
21	or/14-20	735	Advanced	Display Results | More	
22	11 not 21	1523	Advanced	Display Results | More	
23	exp Immune System Diseases/	1408288	Advanced	Display Results | More	
24	limit 23 to (abstracts and last 10 years)	336371	Advanced	Display Results | More	
25	(jama pediatrics or "archives of pediatrics & adolescent medicine").jn.	6634	Advanced	Display Results | More	
26	pediatrics.jn.	35256	Advanced	Display Results | More	
27	archives of disease in childhood fetal & neonatal edition.jn.	3545	Advanced	Display Results | More	
28	journal of pediatrics.jn.	31758	Advanced	Display Results | More	
29	"pediatric research".jn.	11732	Advanced	Display Results | More	
30	24 and (or/25-29)	1039	Advanced	Display Results | More	
31	remove duplicates from 30	1039	Advanced	Display Results | More	
32	limit 31 to (comment or editorial or festschrift or historical article or letter)	3	Advanced	Display Results | More	
33	31 not 32	1036	Advanced	Display Results | More	
34	limit 33 to child	655	Advanced	Display Results | More	

Contract

35	limit 33 to adult	101	Advanced	Display Results | More
36	limit 33 to "qualitative studies (optimized)"	126	Advanced	Display Results | More
37	limit 33 to clinical trial, all	104	Advanced	Display Results | More
38	limit 33 to randomized controlled trial	86	Advanced	Display Results | More
39	limit 33 to systematic reviews	69	Advanced	Display Results | More
40	limit 33 to comparative study	128	Advanced	Display Results | More
41	limit 33 to evaluation studies	19	Advanced	Display Results | More
42	33 and Program Evaluation/	7	Advanced	Display Results | More
43	or/36-42	369	Advanced	Display Results | More
44	33 not 43	667	Advanced	Display Results | More
45	exp Endocrine System Diseases/	930618	Advanced	Display Results | More
46	limit 45 to (abstracts and last 10 years)	265380	Advanced	Display Results | More
47	(jama pediatrics or "archives of pediatrics & adolescent medicine").jn.	6634	Advanced	Display Results | More
48	pediatrics.jn.	35256	Advanced	Display Results | More
49	archives of disease in childhood fetal & neonatal edition.jn.	3545	Advanced	Display Results | More
50	journal of pediatrics.jn.	31758	Advanced	Display Results | More
51	"pediatric research".jn.	11732	Advanced	Display Results | More
52	46 and (or/47-51)	562	Advanced	Display Results | More
53	remove duplicates from 52	562	Advanced	Display Results | More
54	limit 53 to (comment or editorial or festschrift or historical article or letter)	2	Advanced	Display Results | More
55	53 not 54	560	Advanced	Display Results | More
56	limit 55 to child	323	Advanced	Display Results | More
57	limit 55 to adult	83	Advanced	Display Results | More
58	limit 55 to "qualitative studies (optimized)"	37	Advanced	Display Results | More
59	limit 55 to clinical trial, all	39	Advanced	Display Results | More
60	limit 55 to randomized controlled trial	29	Advanced	Display Results | More
61	limit 55 to systematic reviews	17	Advanced	Display Results | More
62	limit 55 to comparative study	59	Advanced	Display Results | More
63	limit 55 to evaluation studies	8	Advanced	Display Results | More
64	55 and Program Evaluation/	1	Advanced	Display Results | More
65	or/58-64	135	Advanced	Display Results | More
66	55 not 65	425	Advanced	Display Results | More
67	exp Gastrointestinal Diseases/	911300	Advanced	Display Results | More
68	limit 67 to (abstracts and last 10 years)	231241	Advanced	Display Results | More
69	(jama pediatrics or "archives of pediatrics & adolescent medicine").jn.	6634	Advanced	Display Results | More
70	pediatrics.jn.	35256	Advanced	Display Results | More
71	archives of disease in childhood fetal & neonatal edition.jn.	3545	Advanced	Display Results | More
72	journal of pediatrics.jn.	31758	Advanced	Display Results | More
73	"pediatric research".jn.	11732	Advanced	Display Results | More
74	68 and (or/69-73)	741	Advanced	Display Results | More
75	remove duplicates from 74	741	Advanced	Display Results | More
76	limit 75 to (comment or editorial or festschrift or historical article or	2	Advanced	

	letter)			Display Results | More
77	75 not 76	739	Advanced	Display Results | More
78	limit 77 to child	324	Advanced	Display Results | More
79	limit 77 to adult	45	Advanced	Display Results | More
80	limit 77 to "qualitative studies (optimized)"	44	Advanced	Display Results | More
81	limit 77 to clinical trial, all	77	Advanced	Display Results | More
82	limit 77 to randomized controlled trial	62	Advanced	Display Results | More
83	limit 77 to systematic reviews	50	Advanced	Display Results | More
84	limit 77 to comparative study	81	Advanced	Display Results | More
85	limit 77 to evaluation studies	8	Advanced	Display Results | More
86	77 and Program Evaluation/	0	Advanced	Save | More
87	or/80-86	227	Advanced	Display Results | More
88	77 not 87	512	Advanced	Display Results | More
89	exp Respiratory Tract Diseases/	1275181	Advanced	Display Results | More
90	limit 89 to (abstracts and last 10 years)	315717	Advanced	Display Results | More
91	(jama pediatrics or "archives of pediatrics & adolescent medicine").jn.	6634	Advanced	Display Results | More
92	pediatrics.jn.	35256	Advanced	Display Results | More
93	archives of disease in childhood fetal & neonatal edition.jn.	3545	Advanced	Display Results | More
94	journal of pediatrics.jn.	31758	Advanced	Display Results | More
95	"pediatric research".jn.	11732	Advanced	Display Results | More
96	90 and (or/91-95)	1798	Advanced	Display Results | More
97	remove duplicates from 96	1798	Advanced	Display Results | More
98	limit 97 to (comment or editorial or festschrift or historical article or letter)	8	Advanced	Display Results | More
99	97 not 98	1790	Advanced	Display Results | More
100	limit 99 to child	720	Advanced	Display Results | More
101	limit 99 to adult	130	Advanced	Display Results | More
102	limit 99 to "qualitative studies (optimized)"	154	Advanced	Display Results | More
103	limit 99 to clinical trial, all	267	Advanced	Display Results | More
104	limit 99 to randomized controlled trial	230	Advanced	Display Results | More
105	limit 99 to systematic reviews	129	Advanced	Display Results | More
106	limit 99 to comparative study	263	Advanced	Display Results | More
107	limit 99 to evaluation studies	38	Advanced	Display Results | More
108	99 and Program Evaluation/	15	Advanced	Display Results | More
109	or/102-108	710	Advanced	Display Results | More
110	99 not 109	1080	Advanced	Display Results | More

Save Remove Combine with: AND OR

Save All Edit Create RSS View Saved

Advanced Search | Basic Search | Find Citation | Search Tools | Search Fields | Multi-Field Search

1 Resource selected | Hide | Change
